# Precision micro-mechanical components in single crystal diamond by deep reactive ion etching

**DOI:** 10.1038/s41378-018-0014-5

**Published:** 2018-06-18

**Authors:** Adrien Toros, Marcell Kiss, Teodoro Graziosi, Hamed Sattari, Pascal Gallo, Niels Quack

**Affiliations:** 1 EPFL STI IMT GR-QUACK, Station 11, CH-1015 Lausanne, Switzerland; 2 LakeDiamond SA, Rue Galilée 7, CH-1400 Yverdon-les-Bains, Switzerland

## Abstract

The outstanding material properties of single crystal diamond have been at the origin of the long-standing interest in its exploitation for engineering of high-performance micro- and nanosystems. In particular, the extreme mechanical hardness, the highest elastic modulus of any bulk material, low density, and the promise for low friction have spurred interest most notably for micro-mechanical and MEMS applications. While reactive ion etching of diamond has been reported previously, precision structuring of freestanding micro-mechanical components in single crystal diamond by deep reactive ion etching has hitherto remained elusive, related to limitations in the etch processes, such as the need of thick hard masks, micromasking effects, and limited etch rates. In this work, we report on an optimized reactive ion etching process of single crystal diamond overcoming several of these shortcomings at the same time, and present a robust and reliable method to produce fully released micro-mechanical components in single crystal diamond. Using an optimized Al/SiO_2_ hard mask and a high-intensity oxygen plasma etch process, we obtain etch rates exceeding 30 µm/h and hard mask selectivity better than 1:50. We demonstrate fully freestanding micro-mechanical components for mechanical watches made of pure single crystal diamond. The components with a thickness of 150 µm are defined by lithography and deep reactive ion etching, and exhibit sidewall angles of 82°–93° with surface roughness better than 200 nm rms, demonstrating the potential of this powerful technique for precision microstructuring of single crystal diamond.

## MEMS: fast and precise diamond etching

Deep reactive ion etching of single crystal diamond enables precise fabrication of freestanding micro-mechanical components. Diamond is an attractive material for nano- and microscale components due to its high hardness and stiffness, coupled with low mechanical friction. However, reactive ion etching of diamond is challenging due to several limitations of existing techniques, including shallow etch depths and rough surfaces. Now, a team led by Niels Quack at EPFL in Switzerland report a deep reactive ion etching process that utilizes a three-layer Al/SiO_2_ hard mask that yields etch rates >30 µm/h and precise dimensional control of features. They demonstrate released single crystal diamond watch components with a thickness of 150 µm and surface roughness better than 200 nm. This new approach paves the way toward high-volume precise fabrication of diamond components.

## Introduction

In recent years, the growth of synthetic diamond crystals has been industrialized based on high pressure high temperature (HPHT) or chemical vapor deposition (CVD) growth techniques^1,2^. Several suppliers provide today commercial offerings of high-quality single crystal diamond substrates, cut and polished to plates in the size of a few tens of square millimeters and up to several hundreds of microns thick (e.g., Element Six or LakeDiamond SA). Such plates are of uniform size and free from defects typically present in natural diamonds, which make them ideal substrates for optical and mechanical components made entirely of single crystal diamond.

Traditional techniques for structuring of diamond crystals typically involve cutting and fine polishing^3^. While these techniques allow for flat surfaces of optically superb quality, they can be applied to curved structures and microstructures only to a limited extent. The most advanced technique for precision structuring of single crystal diamond today is ultrashort pulse laser microstructuring, which has recently been performed on such substrates and allows to produce up to 1.2 mm-thick curved optical components with a precision of 10 µm and a surface roughness of 1 µm rms^4^. However, the dimensions of these substrates and their excellent uniformity also allow them to be processed using standard microfabrication techniques, which open an entirely novel direction of processing methods and a potential for parallelization. Consequently, the availability of such substrates has led to a tremendous increase in interest in scientific and engineering research over the past decade, targeting to exploit the extraordinary optical, mechanical, and thermal properties of this unique material.

Examples of recent demonstrations in single crystal diamond micro- and nanosystem engineering include mechanical systems such as high-Q nanomechanical resonators^5^, extremely hard nanowire tips^6,7^, nanoindenters^8^, and stiff cantilevers^9^, and optical components such as micro-lenses^10,11^, gratings^12^, and microcavities^13^. In these applications, single crystal diamond permits excellent performance compared to similar structures made in any other material. Enormous interest is further arising of the intriguing possibilities to include optically active defects, color-centers such as the nitrogen-vacancy complex, which have led to entirely novel applications, such as scanning diamond magnetometry^14,15^, on-chip quantum information processing^16^, labeling^17,18^, and quantum cryptography^19,20^ (for a recent review on diamond nanofabrication, see ref. ^21^).

Precision structuring of diamond has—in several of these demonstrations—been achieved using microfabrication based on lithography and reactive ion etching. The advantages of such a fabrication approach lie in the high resolution allowed by lithography, the possibility of parallel fabrication, and the smooth surfaces^22^ that can be obtained by reactive ion etching processes.

For devices on the nanoscale range, electron beam lithography is routinely used to pattern e-beam resists such as hydrogen silsesquioxane. Once patterned, these resists are suitable etch masks for the reactive ion etching of diamond when limited etching depths are required. Lončar et al.^7^ have used this approach to fabricate single crystal diamond nanowires of about 2 µm in height. For applications requiring deeper etching, structured electron beam resists commonly serve as etch mask to pattern underlying metallic or dielectric layers, which in turn serve as hard mask for the reactive ion etching of diamond to achieve more important etch depths. Diamond nanoslabs were demonstrated by Englund et al.^23^ using ZEP electron beam resist to pattern a 80 nm-thick chromium hard mask, subsequently etching the diamond substrate 10 µm deep. Finally, patterned electron beam resists are also suitable for lift-off processes. Gu et al.^24^ obtained diamond pillars about 1 µm tall by evaporating chromium on an electron beam-patterned PMMA resist, followed by lift-off and reactive ion etching of diamond. While these methods use electron beam lithography and allow obtaining nanostructures, photolithography is an excellent alternative when the desired critical dimensions are in the micrometer range. For instance, extensive developments in the field of diamond micro-lenses have been reported, which generally are based on photolithography and thermal reflow for patterning a photoresist layer. Wang et al.^25^ have recently demonstrated micro-lenses with diameters of about 15 µm and height of about 300 nm. For such etching depths and similarly to electron beam resists, photoresists are generally well suited as etch mask material during the reactive ion etching of diamond. The vast majority of etching processes are based on oxygen plasma in combination with a secondary gas. Pure oxygen plasma has been used by Degen et al.^6^ to produce diamond tips of a few micrometers in length, while Maletinsky et al.^15^ use an argon/oxygen plasma to fabricate 2 µm long pillars on a thin diamond membrane, obtained beforehand with cycles of pure oxygen and argon/chlorine plasmas. Etching processes can further be tailored to specific requirements by modifying parameters such as the chamber pressure, the gas flows, the plasma, and the bias power. These adjustments influence the directionality of the etching, the etched surface roughness, the etching rate, and the selectivity to the hard mask. Etch rates up to 40 µm/h^26^ and selectivity to the hard mask material up to 1:200 have previously been reached^27^, however, structures etched into single crystal diamond have typically been limited to a thickness ranging from a few hundreds of nanometers to a few tens of microns. The highest hitherto reported etch depth amounts to 55 µm using a high-grade steel structure as a hard mask, positioned by pick and place on the diamond surface^28^. However, this approach does not benefit from the precision of photolithography techniques. To the best of our knowledge, the structure with the most important thickness realized in single crystal diamond by photolithography and reactive ion etching techniques is a 50 µm-thick diamond probe recently demonstrated by Yacoby et al.^29^ The structure is obtained by photolithography and a pure oxygen plasma with a titanium hard mask, subsequently mechanically released from the diamond substrate and attached to a tipless atomic force microscopy (AFM) cantilever. This thickness range is well suited for the fabrication of released diamond devices for applications such as cantilevers for AFM; however, for micro-mechanical components such as required in time-keeping mechanisms in mechanical watches, there is a need for a minimum threefold increase in etch depth.

This limitation in etch depth arises from a combination of challenges associated to the requirements for deep reactive ion etch processes: in order to achieve high anisotropic etch rates and vertical etch profiles, typically high-density plasmas with high platen bias power are required. While the high energy from the platen bias results in increased etch rate, the hard mask is equally subjected to high-energy ion impacts. Thus, the hard mask is rapidly consumed, consequently a thick hard mask layer is required to achieve deep etching in diamond. Furthermore, sputtering of the hard mask material with subsequent re-deposition on the diamond surface can occur. With the re-deposited particles acting locally as hard mask material, further etching is prevented at these locations and columnar whisker structures are formed. This effect will result in rough surfaces^30^ and will lead to slow down and eventual stopping of the etch before reaching the bottom of the substrate. This phenomenon is commonly referred to as micromasking, which intensifies with increasing etch duration and plasma anisotropy^12^, thus making it difficult to reach high etch depths. An additional practical challenge is associated to the substrate size being limited to a few tens of mm^2^: when applying standard spin coating for photoresist deposition, edge-bead formation of the photoresist^31–33^ will limit the resolution of the lithography process.

## Materials and Methods

**Fig. 1:**
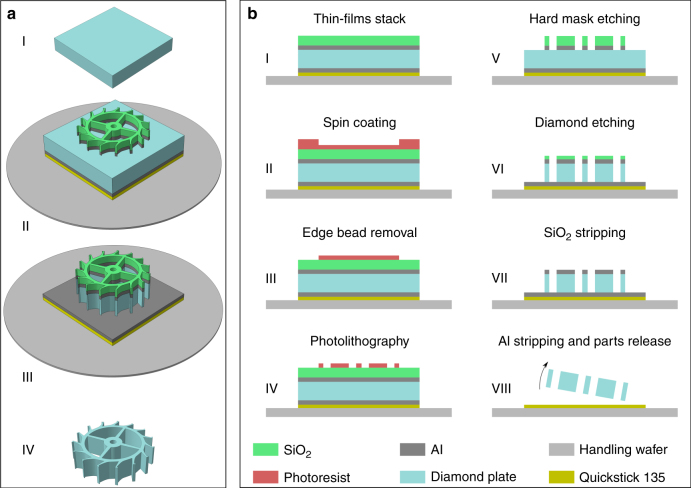
Microfabrication process for freestanding components in single crystalline diamond. **a** Schematic 3D representation of the main microfabrication process steps for the fabrication of released components in single crystalline diamond. On the top surface of the initial 5.5 mm × 5.5 mm × 150 µm diamond substrate (step I), a thick SiO_2_ hard mask on an Al adhesion layer is patterned by photolithography and dry etching. A sacrificial Al layer is deposited on the diamond backside and the stack is assembled on a handling wafer with a mounting wax (step II). The pattern is transferred to the diamond by deep reactive ion etching (step III). Finally, the remaining hard mask and the sacrificial Al layer on the bottom surface of the diamond are stripped to release the parts (step IV). **b** Detailed cross-section schematic of the microfabrication process steps for the fabrication of released components in single crystalline diamond showing the thin-film stack deposition (step I), the photoresist spin coating with the edge-bead formation (step II) and subsequent removal (step III), the photolithography with the components patterns (step IV), the hard mask etching (step V), the diamond deep reactive ion etching (step VI), the remaining hard mask stripping (step VII), and the Al stripping to release the parts (step VIII)

In this work, we have developed a method to overcome these limitations to reach unprecedented depths in single crystalline diamond, allowing to completely etch through diamond substrates and releasing lithographically defined components. The fabrication process is shown schematically in Fig. 1, and the detailed protocol is described in Section I of the supplementary information.

In a first step, a suitable hard mask is deposited on the diamond substrate with a typical dimension of 5.5 mm × 5.5 mm × 150 µm. Metal thin films such as aluminum have previously been used as hard mask materials for single crystal diamond etching. They provide good adhesion on diamond^33^, and show good selectivity (~1:100)^27^. However, metal hard masks generally result in significant micromasking during highly energetic oxygen plasma-based diamond etching^30,34^. In contrast, dielectric thin films of silicon oxide (SiO_2_) or alumina (Al_2_O_3_) have been used previously with minimum micromasking effects in pure oxygen plasmas^27^. In this work, several hard mask materials (Al, Si, Al_2_O_3_, and SiO_2_) were experimentally assessed using various plasma parameters and compositions. A pure oxygen plasma combined with a SiO_2_ hard mask strongly reduced micromasking effects while exhibiting a high etch rate and a good selectivity to the diamond. However, challenges arose from the thick layer of hard mask needed to etch through the diamond and the low adhesion of silicon oxide layers on diamond^33^.

With the oxygen plasma parameters and the SiO_2_ hard mask used in our process, a selectivity of 1:50 was measured. This would require a 3 µm-thick SiO_2_ hard mask to etch through a 150 µm-thick diamond plate. However, when using a highly biased plasma, hard mask faceting is a commonly encountered issue that leads to angled sidewalls in the etched substrate^35^. A well-known method used to minimize the effects of the hard mask faceting is to use a hard mask thicker than required. In our process, a 7 µm-thick hard mask is used, which is much thicker than usually encountered in common microfabrication processes. This poses a number of challenges in terms of mask adhesion and quality, but also in terms of mask patterning: the low adhesion can result in delamination of the hard mask layer during lithography^33^ or when exposed to the plasma. Moreover, SiO_2_ layers with such thicknesses are generally highly susceptible to cracking due to the large internal stress^36^. To avoid the mask from delaminating during the photolithography (specifically when the photomask is removed from the photoresist-coated diamond substrate surface after the exposure, or during the development of the photoresist), an adhesion layer is needed between the diamond and the hard mask. Chromium or titanium are typical adhesion layer materials, however in our process, a 200 nm-thick Al layer resulted in optimum adhesion of the hard mask to the diamond substrate. In addition, an identical Al layer was deposited on the backside of the diamond plate. This sacrificial layer is used for the removal of the etched components when the diamond through etch is completed.

The SiO_2_ hard mask was deposited by radio frequency sputtering. Sputtering in Ar with added O_2_ is beneficial for decreasing the stress and the roughness in the film, while increasing its hardness and adhesion^37^. However, the addition of O_2_ also leads to a very slow deposition rate, around five times slower than when using only Ar. Therefore, a first thin (65 nm) layer of SiO_2_ was deposited with both Ar and O_2_ flow, while the remaining 7 µm layer was deposited without added O_2_ to obtain an increased deposition rate. The thin layer deposited under oxygen flow acts effectively as a buffer layer between the Al and the SiO_2_ layer deposited without oxygen.

**Fig. 2:**
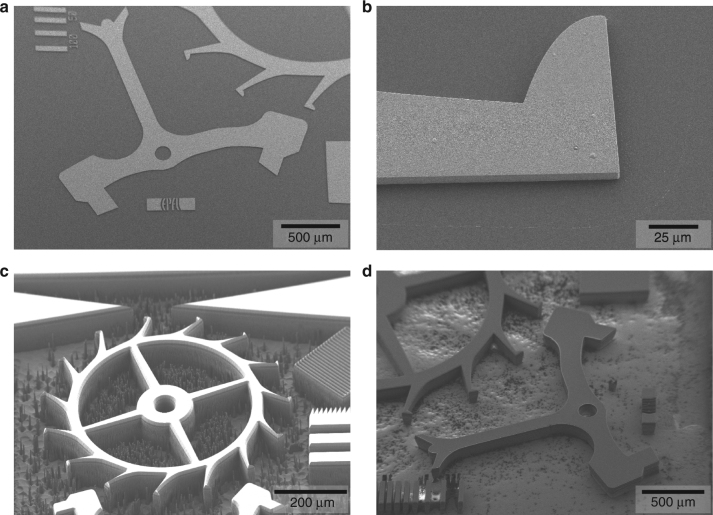
Microfabrication process development for freestanding components in single crystalline diamond. **a** High-quality thin-film hard mask layer stacks (200 nm Al and 7 µm SiO_2_) deposited on diamond with **b** high-resolution patterns. While initial experiments with Al hard masks resulted in **c** strong micromasking after 4 h of Ar/O_2_ reactive ion etching, this effect was **d** strongly reduced by the use of the SiO_2_ hard mask, and 150 µm-thick diamond substrates could be fully etched through after 5 h O_2_ reactive ion etch

For patterning the hard mask, the diamond plate was temporarily attached to a 4-inch handling Si wafer with a mounting wax to allow compatibility with standard microfabrication tools (Fig. 1b, step I). A 2.5 µm-thick layer of photoresist was spin coated on the diamond plate surface, which leads to an important edge bead (Fig. 1b, step II) that has to be removed, in order to have a close contact between the photomask and the photoresist during exposure for high-resolution pattern transfer. Several solutions have previously been employed for edge-bead reduction or removal, including equalizing height^32,38^, in hole placement^39,40^, mask pick and place^41^, and stamp transfer^34,42^. We here developed a two-cycle photolithography exposure approach to completely remove the edge-bead region on the small substrate (Section II of supplementary information). After the photoresist spin coating, a first exposure is performed on a 0.5 mm-wide frame covering the inside of the four edges of the diamond plate, with a high dose adapted to the important thickness of the edge bead. In a subsequent first development (Section III of supplementary information), the edge bead is removed (Fig. 1b, step III) and allows to perform a closely contacted exposure of the central region using a photomask with the component pattern. A second development completes the photolithography process (Fig. 1b, step IV) and is followed by the dry etching of the SiO_2_ hard mask, a short dip in buffered HF to smoothen the SiO_2_ sidewalls (Section IV of supplementary information), and the dry etching of the Al adhesion layer (Fig. 1b, step V). It is essential to perform the SiO_2_ hard mask etching by iterating reactive ion etch and cool-down steps, as the thermal conductance between the photoresist layer and the cooled substrate holder is lowered by the wafer-adhesive-diamond stack. Without cool down between etching steps, the photoresist will overheat and disintegrate before the completion of the hard mask etch. This microfabrication process flow using a hard mask layer stack of 200 nm Al and 7 µm SiO_2_, and a two-cycle photolithography for edge-bead removal, resulted in well-resolved and defect-free hard mask layers on 5.5 mm × 5.5 mm × 150 µm single crystal diamond substrates, as shown in Fig. 2a, b.

The diamond etching (Section V of supplementary information) is performed in an inductively coupled plasma module (Fig. 1b, step VI), and is followed by the remaining SiO_2_ hard mask stripping (Fig. 1b, step VII) and sacrificial Al stripping to release the parts (Fig. 1b, step VIII). While initial experiments with Al hard masks resulted in excessive micromasking after 4 h of Ar/O_2_-based reactive ion etching (Fig. 2c), this effect was strongly reduced by the use of the SiO_2_ hard mask (Fig. 2d), and 150 µm-thick diamond substrates could be fully etched through after 5 h O_2_ reactive ion etch.

## Results and discussion

**Fig. 3:**
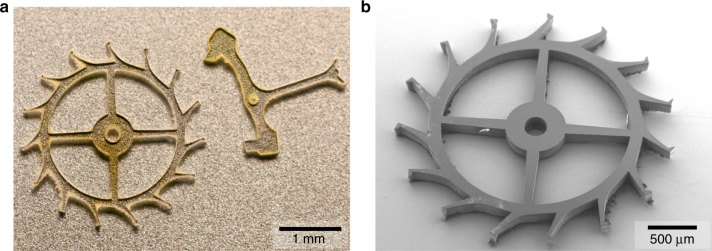
Micromechanical components in single crystal diamond. **a** Photograph of a 150 µm-thick escape wheel and anchor, obtained from a single crystal diamond by deep reactive ion etching. **b** Scanning electron microscope recording of the escape wheel, highlighting the unprecedented precision of the single crystal diamond component as a result from the microfabrication process

In order to demonstrate the capabilities of the microfabrication process, we chose to manufacture mechanical parts that are central components in time-keeping mechanisms of mechanical watches, including escape wheel and anchor. Nevertheless, the process is suitable for the fabrication of a wide range of possible components, such as hairsprings, nanoindenters, mechanical tips, diamond blades, optical components, etc. The application for mechanical watches is a domain of particular interest, since the material properties of single crystalline diamond can provide low-weight components, high-energy storage, extremely low coefficient of friction^43^, amagnetic mechanisms, and attractive visual appeal. The application typically requires micrometer dimensional tolerances with low sidewall roughness. Figure 3a shows a photograph of two fabricated components, an escape wheel, and corresponding anchor. The yellow hue originates from nitrogen impurities in the particular HPHT single crystalline diamond substrate that was used for the process. It is possible to obtain colorless components or fancy colors (e.g., blue or pink) by using CVD diamond, with a high level of purity or by deliberately adding specific impurities during growth or irradiating the substrate after growth. Figure 3b shows a scanning electron microscope (SEM) recording of the escape wheel. The image reveals the precise definition of fine features and good verticality of the sidewalls.

Based on optical microscope inspection, patterns measured on the diamond part top surface were laterally reduced on each edge by 5.7 µm (i.e., total reduction of 11.4 µm) in size compared to the original photomask design. This reduction in feature size is resulting from a combination of contact lithography and hard mask recess during the etch process, and can be further reduced by including an appropriate mask bias^27^.

**Fig. 4:**
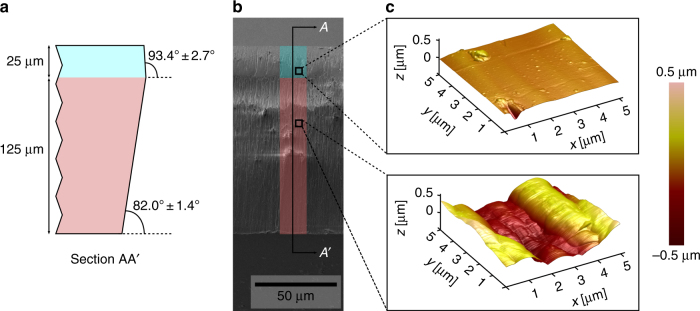
Sidewall profile and surface roughness of micromechanical components in single crystal diamond. **a** Schematic cross-sectional view of the released parts’ sidewalls. **b** Scanning electron microscope recording of a sidewall after deep reactive ion etch, revealing two distinct regions separated at 25 µm from the top edge. Atomic force microscope measurements show a **c** surface roughness as low as 20 nm rms in the top region, while **d** the bottom region exhibits a surface roughness of 200 nm rms. The variation in roughness can be attributed to the different sidewall profiles in the top and the bottom region

The profile of the sidewall (Fig. 4a) is composed of two distinct regions. The bottom region (extending to about 125 µm from the bottom edge) exhibits a negative tapered (i.e., “retrograde”) profile and an angle of 82.0° ± 1.4°, while the top region (extending to about 25 µm from the top edge) shows a positive tapered profile and a 93.4° ± 2.7° angle. The angle measurement method is described in Section VI of the supplementary information. The positive profile of the top region originates from the hard mask recess during the etching^44^, while we attribute the origin of the bottom region negative profile to an isotropic component of the plasma characteristics, which can be further optimized by adjusting the plasma etch parameters.

While this sidewall profile can already prove sufficient for mechanical watch components, we expect that the verticality can further be improved by adjusting the combination of process pressure and bias power. Similar optimizations have been demonstrated in single crystal diamond for etch depths of up to 10 µm, where sidewall angles of 88.45° have been achieved by using periodical renewing of the hard mask during the etch^34^. Alternative approaches for sidewall verticality improvement can include cyclic passivation of the sidewalls as known from silicon deep reactive ion etching (Bosch process). In silicon, sidewall angles of 89.7° have been demonstrated for similar etching depths of 130 µm^45^. Such quasi-ideal sidewall angles are obtained by adjusting the etching time to passivation time ratio^46^. While similarly robust sidewall passivation techniques have not yet been developed for the deep etching of diamond, possible routes for sidewall passivation mechanisms for diamond etching can include controlled re-deposition using Ni or Ni–Ti alloy hard masks^47^.

The sidewall roughness of the components was measured by AFM. Figure 4b shows a SEM recording of the inspected sidewall, and the corresponding AFM measurements for two selected sites in Fig. 4c, d. The top region of the sidewall exhibits a surface roughness as low as 20 nm (rms), while the bottom region shows a surface roughness of about 200 nm (rms). These values correspond to the standard deviation of the height in 5 µm × 5 µm areas, after plane fitting (i.e., data centering and tilt removal). We attribute the two regions of different roughness to the different profiles of the top and bottom region of the sidewall. While the bottom region retains striations caused by the original hard mask sidewalls roughness, the top region is submitted to a continuous smoothening as the etch progresses. This top region smoothening effect is in agreement with previous reports on reactive ion etching of diamond^44^.

The surface quality obtained with our deep reactive ion etching process constitutes an improvement of >5× (200 nm rms region) and >50× (20 nm rms region) compared to surface roughness reported on sidewalls of 1.2 mm single crystalline diamond components obtained by femtosecond laser structuring^4^. For much lower etch depths of up to 1 µm, previous reports have shown even smoother sidewalls in single crystal diamond by reactive ion etching^48^, with peak-to-peak values of 10–20 nm (here 400–800 nm), opening an avenue for further improvement of the surface roughness.

While mechanical components will benefit from reduced friction, the reduction in surface roughness opens further possibilities to manufacture optical components such as lenses, gratings, prisms, filters, or optical windows.

## Conclusion

In conclusion, we have for the first time demonstrated fully released precision micro-mechanical components in 150 µm-thick single crystalline diamond, obtained by deep reactive ion etching. Prototypes of escape wheels and anchors for mechanical watches were successfully fabricated. Commonly encountered challenges in deep reactive ion etching of diamond were overcome by an optimized hard mask deposition and patterning process in combination with a high-density oxygen plasma etch with high bias power, avoiding micromasking while obtaining high etch rate (30 µm/h) and high selectivity to the hard mask (1:50) at the same time. The hard mask consists of a three-layer stack to provide a well-adhering and defect-free thick hard mask for complete diamond through etch. An Al adhesion layer avoids the cracking or delamination of the thick SiO_2_ hard mask, which is deposited in two steps. In addition, a two-step photolithography procedure was used as effective photoresist edge-bead removal method, allowing for a high-fidelity photolithography process. A sacrificial Al layer between the diamond and the handling wafer allows for a simple release of the fabricated components when the etching is completed. The characterization of the fabricated parts revealed sidewalls angles of 82°–93° and sidewalls roughness of better than 200 nm rms, at least 5× better than surface roughness obtained with traditional ultrashort pulse laser microstructuring techniques. This novel fabrication method is fully compatible with standard thin-film deposition techniques, and photolithography allows to define arbitrarily shaped components for novel designs and geometries with high yield and high resolution. While the size of currently available single crystal diamond substrates is still limited, the process allows for parallelization of the critical fabrication steps, including etching and release, by multi-chip placement on a single carrier wafer, providing a route for volume production of lithographically defined precision micro-mechanical components.

## Electronic supplementary material

Detailed Description of Experimental Procedures
