# Supplementary material for: Precision micro-mechanical components in single crystal diamond by deep reactive ion etching
Source: Microsyst Nanoeng. 2018 Jun 18;4:12. doi: 10.1038/s41378-018-0014-5 (PMC6161503; doi:10.1038/s41378-018-0014-5)
Supplement: Supplementary file 1 — Detailed Description of Experimental Procedures [file 41378_2018_14_MOESM1_ESM.pdf]

Precision micro-mechanical components in single crystal diamond by deep reactive ion etching

Supplementary information

Adrien Toros, Marcell Kiss, Teodoro Graziosi, Hamed Sattari, Pascal Gallo and Niels Quack**

Authors' Affiliations and Addresses:

* Adrien Toros
EPFL STI IMT GR-QUACK
ELG 232, Station 11
1015 Lausanne
Switzerland
Email: adrien.toros@epfl.ch

Marcell Kiss
EPFL STI IMT GR-QUACK
ELG 232, Station 11
1015 Lausanne
Switzerland
Email: marcell.kiss@epfl.ch

Teodoro Graziosi
EPFL STI IMT GR-QUACK
ELG 232, Station 11
1015 Lausanne
Switzerland
Email: teodoro.graziosi@epfl.ch

Dr. Hamed Sattari
EPFL STI IMT GR-QUACK
ELG 232, Station 11
CH-1015 Lausanne
Switzerland
Email: hamed.sattari@epfl.ch

Dr. Pascal Gallo
LakeDiamond SA
Rue Galilée 7
1400 Yverdon-les-Bains
Switzerland
Email: pascal.gallo@lakediamond.ch
Web: www.lakediamond.ch

* Prof. Niels Quack
SNSF Assistant Professor
EPFL STI IMT GR-QUACK
ELG 236, Station 11
1015 Lausanne
Switzerland
Phone +41 21 693 73 83
Cell +41 78 604 76 54
Fax: +41 21 693 26 14
Email: niels.quack@epfl.ch
Web: q-lab.epfl.ch

Section I. Components fabrication

The single crystal diamond substrate with dimensions of 5.5 mm x 5.5 mm x 0.15 mm (High Pressure High Temperature (HPHT) raw crystal, grown, cut and polished by LakeDiamond SA) are first cleaned using a Piranha solution (H_2SO_4(96%):H_2O_2(30%) (3:1)). The substrate is then subjected to additional cleaning in an oxygen plasma (600 W, 400 sccm O_2 flow, 0.8 mbar, PVA TePla GIGAbatch) for 2 minutes, and a 200 nm thick Al layer is sputtered on both sides of the substrate immediately afterwards (200 W, 15 sccm Ar flow, 6 min deposition time, Pfeiffer Vacuum SPIDER 600). A 65 nm thick SiO_2 layer is sputtered on the frontside of the substrate under O_2 flow (1000 W, 98 sccm Ar flow, 13 sccm O_2 flow, 4 min deposition time, Pfeiffer Vacuum SPIDER 600), followed by a 7 µm thick silicon oxide layer sputtered without O_2 flow (1000 W, 15 sccm Ar flow, 133 min deposition time, Pfeiffer Vacuum SPIDER 600). The substrate is attached on a silicon handling wafer using QuickStick 135, followed by an Hexamethyldisilazane (HMDS) vapor deposition at 130°C. A 2.5 µm thick layer of ECI 3027 photoresist is spin coated at 1750 rpm, followed by a 5 minutes softbake at 100°C. A first exposure of the photoresist is performed (600 mJ/cm^2, SUSS MicroTec MA6 Gen3) on the edge-bead affected region (from the substrate edge to 0.5 mm inside the substrate), followed by development in AZ 726 MIF developer for 137 seconds. A second exposure (225 mJ/cm^2, SUSS MicroTec MA6 Gen3) is performed on the central region of the substrate, with the pattern of the parts to be fabricated, followed by a development in AZ 726 MIF for 108 seconds. The SiO_2 is etched in a He/H_2/C_4F_8 based plasma in steps with a duration of less than 4 minutes each during a total time of 30 minutes and 20 seconds (1200 W ICP Power, 300 W bias power, 175 sccm He, 30 sccm H_2, 10 sccm C_4F_8, 4 mTorr chamber pressure, SPTS APS). The photoresist is stripped using an O_2 plasma (600 W, 400 sccm O_2 flow, 0.8 mbar, PVA TePla GIGAbatch) for 2 minutes, a 5 minutes immersion in a MICROPOSIT REMOVER 1165 solution heated at 75°C followed by a DI water rinsing and drying under N_2 flow, and a second O_2 plasma (600 W, 400 sccm O2 flow, 0.8 mbar, PVA TePla GIGAbatch) for 2 minutes. In order to smoothen the SiO_2 sidewalls, the substrate is dipped for 15 seconds in a buffered

hydrofluoric acid solution (NH_4F(40%):HF(50%) (7:1)), followed by DI water cleaning and N_2 blow-drying. The aluminum layer is etched in a Cl_2/BCl_3 based plasma for 1 min (800 W coil power, 150 W platen power, 10 sccm Cl_2 flow, 10 sccm BCl_3 flow, 3 mTorr chamber pressure, STS Multiplex ICP), immediately followed by a DI water rinsing and drying under N_2 flow to remove any chlorine residues. The single crystal diamond substrate is etched for approximately 5 hours in an O_2 based plasma (2000 W ICP power, 200 W bias power, 100 sccm O_2 flow, 15 mTorr chamber pressure, SPTS APS) until the Al backside layer is reached, with etch end point determination by visual inspection. The SiO_2 is stripped in an HF (50%) bath, and the Al is etched in an H_3PO_4(85%):CH_3COOH(100%):HNO_3(70%) (83:5.5:5.5) bath at 35°C until it is fully stripped and the parts are released from the handling Si wafer. Finally, the released parts are rinsed in DI water and dried under N_2 flow.

Section II. Photomask design

Two photomasks are used in the fabrication process: the first one (Figure S1a) is used for the edge bead removal and the second (Figure S1b) is used for patterning the components, in particular the presented anchor and escape wheel.

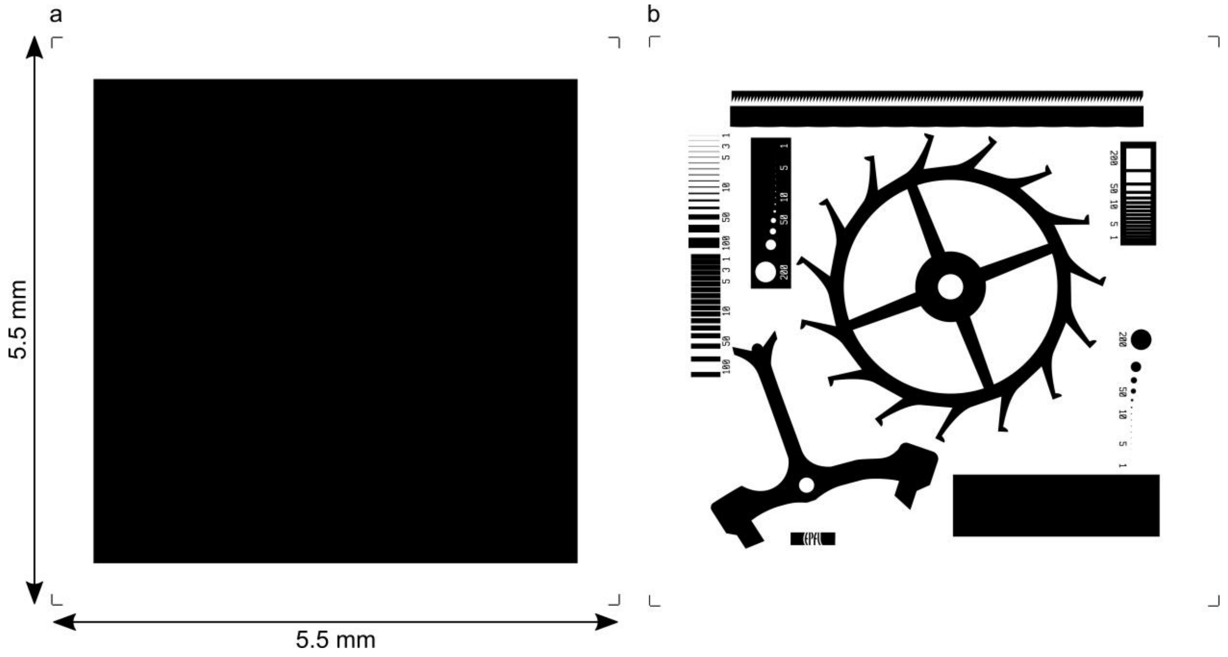

Figure S1 Photomasks design with (a) the edge bead removal pattern and (b) the components patterns.

Section III. Edge Bead Removal

A substantial edge bead is formed after the photoresist spin coating (Figure S2a). This edge bead has to be removed in order to allow a close contact between the photomask and the photoresist during the exposure of the component patterns. Therefore, a first photolithography is performed by exposing the photoresist in a region ranging from the diamond edges to 0.5 mm towards the diamond center, followed by the development of the resist (Figure S2b).

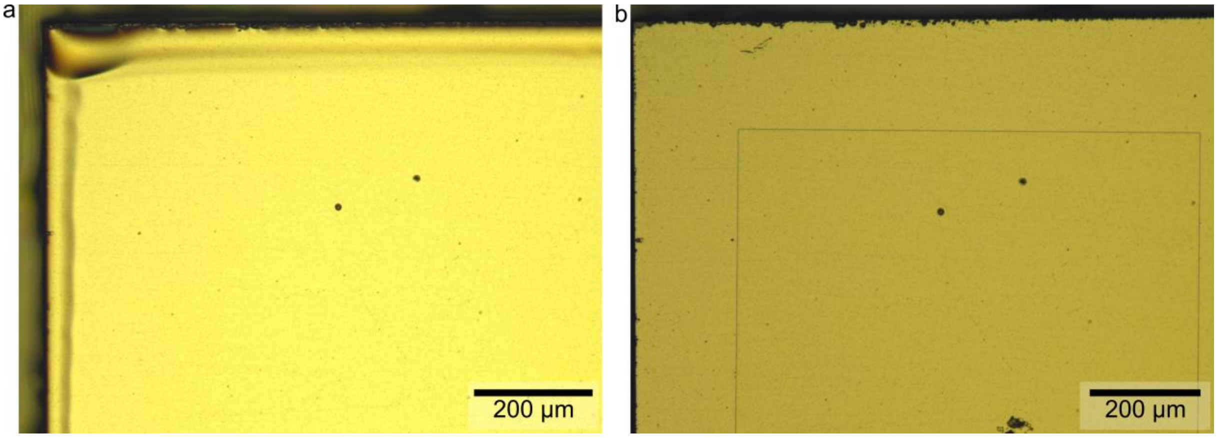

Figure S2 (a) Edge bead observed on a diamond substrate after photoresist coating at 6000 RPM. (b) Diamond substrate surface after edge bead removal, done by exposing the photoresist in a region extending from the diamond edges to 0.5 mm towards the diamond center and developing the photoresist.

Section IV. Hard Mask Sidewall Smoothening

After etching the SiO_2 hard mask, the sidewalls exhibit an important roughness and semi-detached sheets (Figure S3a). As the hard mask topography is transferred to the diamond sidewalls during the etching, it is beneficial to reduce the hard mask roughness before the diamond etching step. This is done by dipping the diamond-on-wafer assembly in a buffered HF bath for 15 seconds, followed by rinsing and drying. The resulting smoothened sidewall is shown in Figure S3b. The exposed Al adhesion layer next to the SiO_2 hard mask is also slightly etched, however this is not problematic since this Al layer has to be removed in the next step by dry etching.

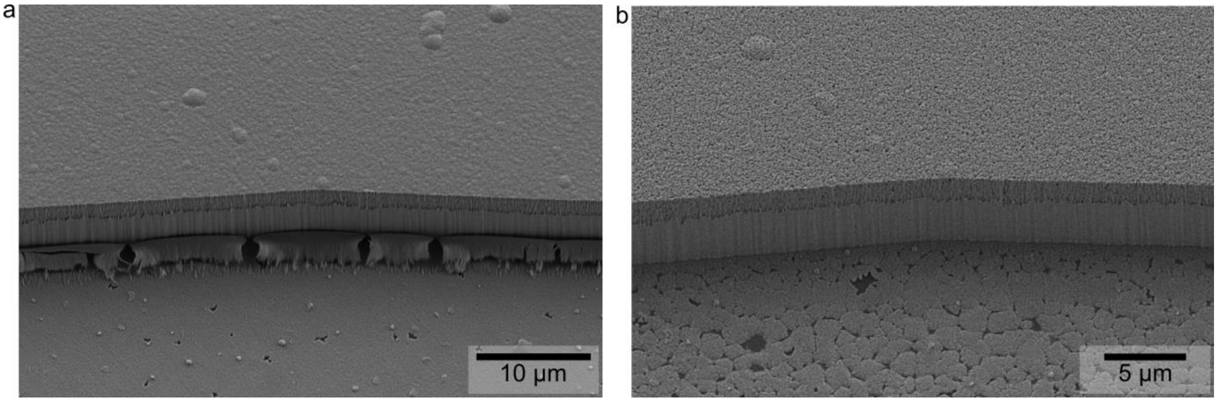

Figure S3 Sidewall of the SiO_2 hard mask before (a) and after (b) the 15 seconds HF dip smoothening step.

Section V. Evolution of the Deep Reactive Ion Etch

Figure S4a shows a 7 µm thick SiO_2 hard mask patterned on a 2.6 mm x 2.6 mm x 0.3 mm single crystalline diamond (Element Six Ltd). The evolution of the deep reactive ion etching performed in an O_2 plasma as described in the experimental section is shown after 1h30 (Figure S4b, etched depth : 48 µm), 2h (Figure S4c, 63 µm), 2h30 (Figure S4d, 79 µm), 4h (Figure S4e, 115 µm) and 5h (Figure S4f, 148 µm), corresponding to an average etch rate of 29.8 µm/hour. In order to use this smaller diamond, a dedicated photomask with smaller dimensions and patterns slightly different from those shown in Figure S1 was fabricated.

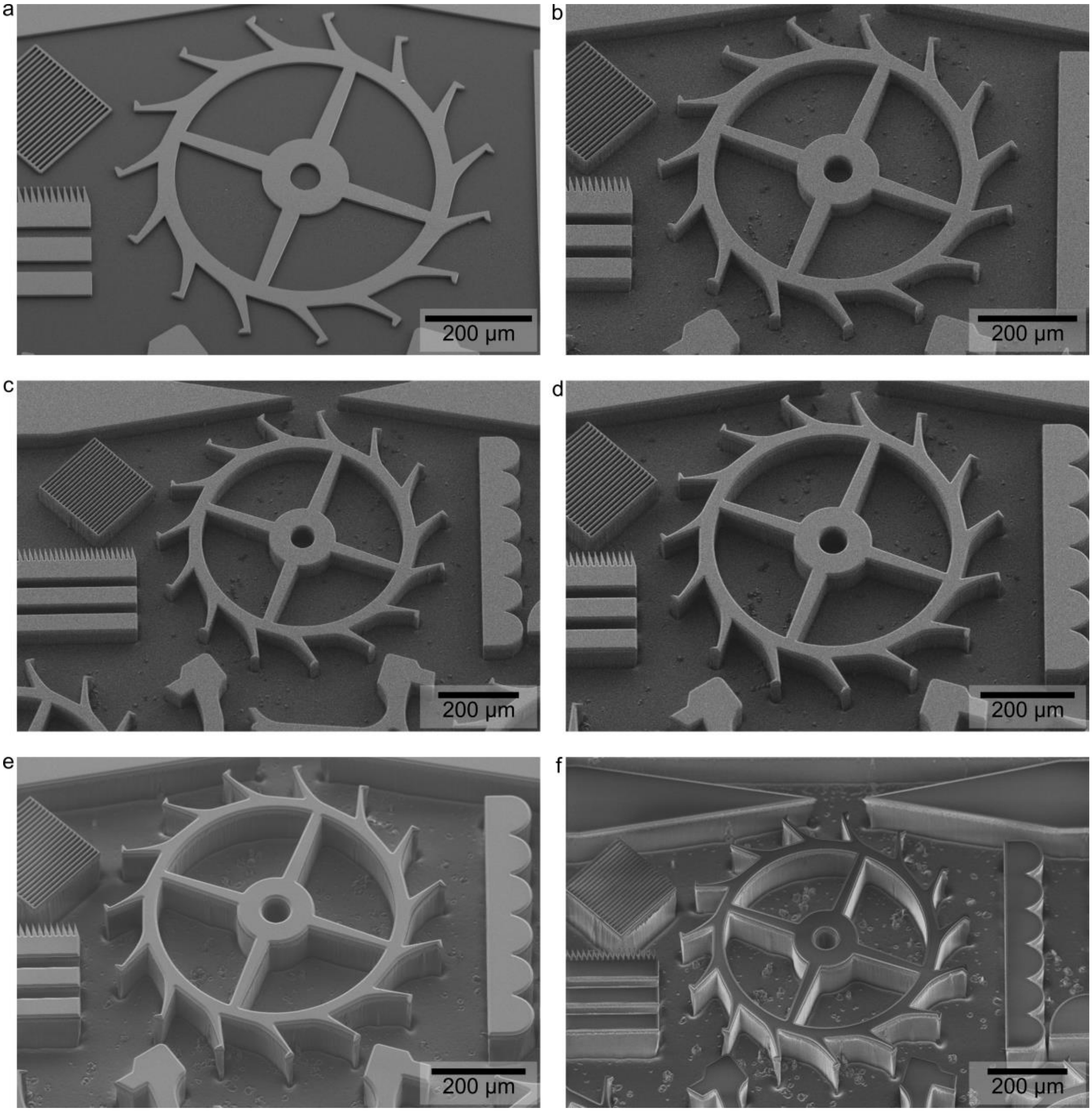

Figure S4 Scanning Electron Microscope recordings of (a) 7 µm thick SiO_2 hard mask patterned on a 2.6 mm x 2.6 mm x 0.3 mm single crystalline diamond substrate, and evolution of the deep reactive ion etch after (b) 1h30, (c) 2h, (d) 2h30, (e) 4h and (f) 5 hours.

Section VI. Sidewall Angle Determination

The slopes of the two sidewall regions were determined by measuring the corresponding angles on the SEM recordings of two sidewalls of a fabricated part (Figure S5), and correcting them to compensate for the 45° tilted stage (e.g. in Figure S5a the lower left angle was measured as 76.8°, corresponding to a sidewall angle of $tan^{-1}\left(\frac{tan\,76.8°}{cos\,45°}\right) = 80.6°$.) The obtained angles (92.8°, 96.1°, 91.1° and 93.7° for the top region and 80.6°, 83.0°, 82.0° and

82.4° for the bottom region) were averaged resulting in angles of 93.4° ± 2.7° (top region) and 82.0° ± 1.4° (bottom region).

These values were confirmed by an independent measurement of the component width at three vertical positions (top edge, bottom edge, and position of largest width) (Figure S5a and S5b) and the corresponding thickness of the two regions of the sidewalls measured from Figure 4b (25 µm for the top region and 125 µm for the bottom region).

For example, from Figure S5b and taking into account the measurements uncertainties, the bottom region angle value and its boundaries were computed as $tan^{-1}\frac{125\ \mu m}{(996\ \mu m-960\ \mu m)/2} = 81.8° < tan^{-1}\frac{125\ \mu m}{(995\ \mu m-965\ \mu m)/2} = 83.2° < tan^{-1}\frac{125\ \mu m}{(994\ \mu m-970\ \mu m)/2} = 84.5°$. The obtained angles (91.1°, 93.4° and 95.7° for the top region and 80.5°, 82.3°, 84.1°, 81.8°, 83.2° and 84.5° for the bottom region) were averaged, resulting in angles of 93.4° ± 2.3° (top region) and 82.7° ± 2.2° (bottom region).

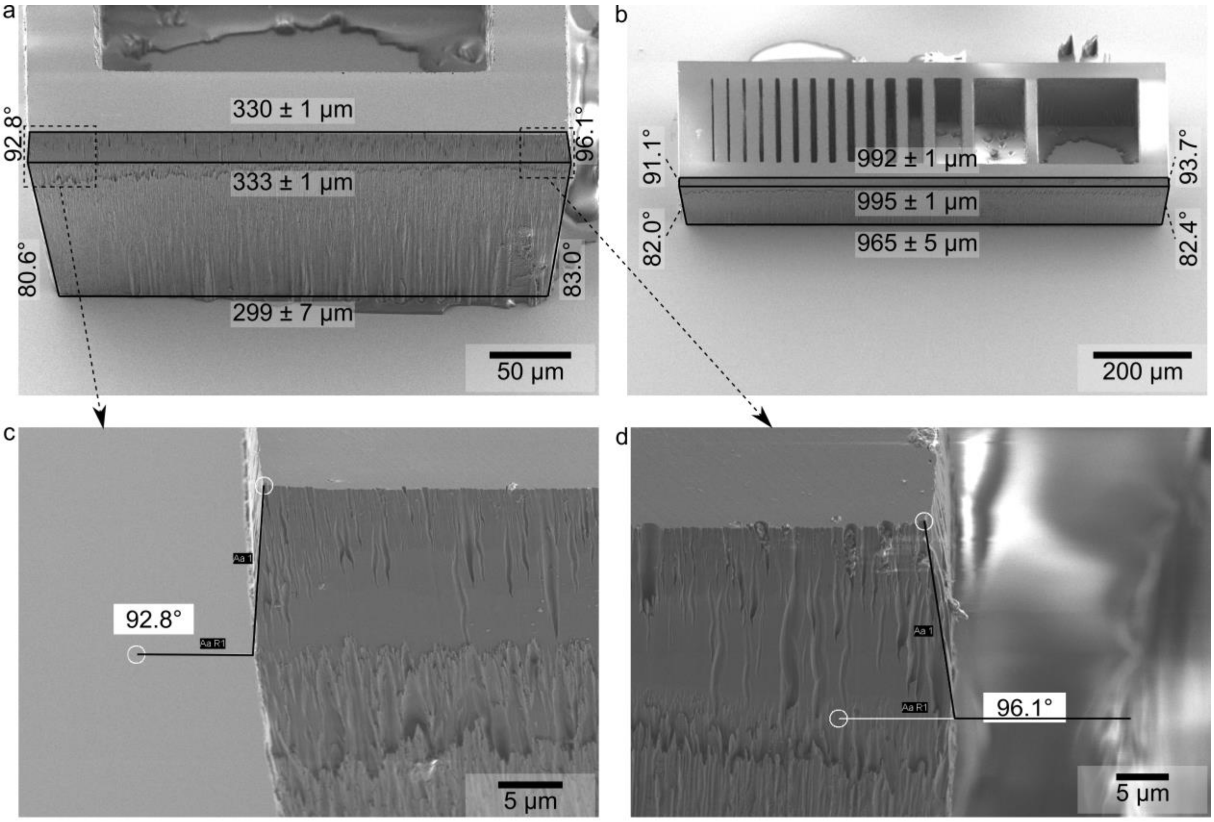

Figure S5 Scanning Electron Microscope recordings of (a, b) two sidewalls of a fabricated part taken with a 45° tilted stage, used for the determination of the sidewall angles. (c, d) Details of the top region sides shown in (a)
